# *ETS-1* and *ETS-2* are upregulated in a transgenic mouse model of pigmented ocular neoplasm

**Published:** 2008-10-29

**Authors:** G. De la Houssaye, V. Vieira, C. Masson, F. Beermann, J.L. Dufier, M. Menasche, M. Abitbol

**Affiliations:** 1 Université Paris-Descartes, EA n°2502 du Ministère de la Recherche, Centre de Recherches Thérapeutiques en Ophtalmologie de la Faculté de Médecine Paris-Descartes-site Necker (CERTO), AP-HP, Hôpital Necker Enfants-Malades, Paris, France; 2 Swiss Institute for Experimental Cancer Research (ISREC), Epalinges, Switzerland

## Abstract

**Purpose:**

Choroidal melanoma is the most common primary malignant ocular tumor in human adults. Relevant mouse models of human uveal melanoma still remain to be developed. We have studied the transgenic mouse strain, Tyrp-1-TAg, to try to gain insight into possible molecular mechanisms common to pigmented ocular neoplasms occurring spontaneously in the eyes of these mice and human choroidal melanoma. The role of two members of the ETS (E26 avian leukemia oncogene) family of transcription factors, ETS-1 and ETS-2, has been investigated in many cancers but has not yet been studied in ocular tumors.

**Methods:**

This is the first study describing the production and distribution of *ETS-1* and *ETS-2* mRNAs and proteins using in situ hybridization and immunohistochemistry in murine ocular tissue sections of normal control eyes and tumoral eyes from mice of the same age. Using semi-quantitative reverse-transcription polymerase chain reaction (RT–PCR) and western blots experiments, we compared changes in *ETS-1* and *ETS-2* expression, their protein levels, and the regulation of some of their target gene expressions at different stages of the ocular tumoral progression in the transgenic mouse model, Tyrp-1-TAg, with those in normal eyes from control mice of the same age.

**Results:**

In normal control adult mouse eyes, ETS-1 was mostly present in the nuclei of all neuroretinal layers whereas ETS-2 was mostly localized in the cytosol of the cell bodies of these layers with a smaller amount present in the nuclei. Both were found in the retinal pigmentary epithelium (RPE). *ETS-1* and *ETS-2* mRNA and protein levels were much higher in the ocular tissues of Tyrp-1-TAg mice than in control ocular tissues from wild-type mice. This upregulation was correlated with tumor progression. We also demonstrated upregulation of *ETS-1* and *ETS-2* target expressions in Tyrp-1-TAg mice when comparing with the same target expressions in control mice.

**Conclusions:**

Our findings suggest that *ETS-1* and *ETS-2* are upregulated in ocular tumors derived from the retinal epithelium and may be involved in one or several signaling pathways that activate the expression of a set of genes involved in ocular tumor progression such as those encoding ICAM-1 (intercellular adhesion molecule-1), PAI-1 (Plasminogen activator inhibitor-1), MCP-1 (monocyte chemoattractant protein-1) and p16 (Cyclin dependent kinase inhibitor 2A).

## Introduction

Simian virus 40 (SV40) large T antigen (T Ag) is a multifunctional, oncoviral protein involved in numerous viral and cellular processes including viral replication, transcriptional activation and repression, blockade of differentiation, and cell transformation [1]. The ability of T Ag to transform cells depends on complex interactions between the viral oncoprotein and various intracellular proteins involved in cell control [2] and transcription regulation such as p53, [3] pRb, and the Rb-related proteins, p107 and p130 [4], and CBP/p300 [5]. The directed expression of SV40 T antigen has led to the development of several important transgenic models with spontaneous epithelial tumor formation. However, one must keep in mind that SV40 large T antigen targets multiple cellular pathways to elicit cellular transformation [6,7]. Unlike cancer arising in the human population, tumors in genetically engineered mouse models arise in mice with well defined genetic backgrounds where genetic variability can be minimized. This offers significant advantages for studying tumor pathogenesis and molecular mechanisms of oncogenesis caused by a single initiating oncogenic event introduced through the mouse germ line.

Choroidal melanoma is the most common primary malignant ocular tumor in human adults. Relevant mouse models of human uveal melanoma still need to be developed. The majority of transgenic lines produced have been generated using the large T SV40 oncogene and either the tyrosinase promoter or the tyrosinase-related promoter-1 promoter [8,9]. Careful analysis suggests that the tumors in these models begin in the neonatal period as a peripapillary multilayered proliferation of retinal pigment epithelial cells. The early tumor cells are characterized by a spindle shape, abundant cytoplasm, round nuclei with uniform staining, and fine granules of melanin pigment [9]. Retinal, choroidal, and optic nerve invasion occurs in 6-10 weeks. By the end of this process, the cells have an appearance similar to human choroidal melanoma cells including increased basophilia, nuclear and cytoplasmic polymorphism, prominent nucleoli, abundant mitosis with tendency to metastasize, and expression of S100 calcium binding protein and Human Melanoma Black (HMB-45) antigens. Tumor growth continues with age and with retinal detachment and extrascleral extension in most murine models [9]. In some instances, the primary tumors seem to originate from the retinal pigmentary epithelium (RPE), and in other instances, they seem to originate from the RPE-choroid interface. It has also been observed in some instances that choroidal tumor formation occurs in the presence of normal RPE. Considering the neuroepithelial origin of RPE and the neural crest origin of choroidal melanocytes, this may be a non-trivial issue when studying the molecular mechanisms of tumorigenesis. The most likely explanation for the differences in transgenic expression is that the RPE is more permissive and/or sensitive to the large T antigen expression than the relatively less active uveal melanocytes.

We studied transgenic mice developing exclusively spontaneous malignant ocular neoplasms without any associated cutaneous melanoma. The transgenic mice that we decided to investigate (Tyrp1-TAg) resulted from the integration of multiple copies into the Y chromosome of an insert with the expression of SV40 large T antigen under the control of the tyrosine-related protein-1 promoter (Tyrp1). This model has been previously described as a model of RPE-derived tumors metastasizing to the brain, inguinal lymph nodes, and spleen [10]. Expression of the SV40 T antigen began at E10.5 and the first abnormalities in the RPE were observed at E15.5. Rapid progression was observed, leading to the development of a single malignant melanocytic tumor in each eye of the transgenic mice and invasion of the choroid. At the age of about two months, the tumor filled the entire eye, and cataracts were present in the anterior chamber. The expression of the SV40 T antigen seemed to be confined to RPE cells. However, several previous studies have shown that early oncogenic sequences of SV40 under transcriptional control of the tyrosinase promoter genetically predispose normal melanocytes to their transformation into malignant melanocytes [8,11-13]. In contrast to normal endogenous *Tyrp1* mRNA levels, transgenic expression levels in neural crest-derived melanocytes is low or below the detection sensitivity threshold. This suggests the absence of important cis-acting regulatory elements favoring significant transcription of the large T antigen coding sequence located within the construct used for producing the transgenic mice that we investigated, Tyrp1-Tag. Indeed, the promoter of the tyrosinase-related family gene, *Tyrp1*, drives detectable transgene expression only in the RPE, even though the gene is also expressed in melanocytes as observed in *Tyrp1* mutant mice [14]. Although the Tyrp1-TAg transgenic mouse model of pigmented ocular neoplasm cannot be strictly considered as a choroidal melanoma, it has many features found in human choroidal melanoma.

The ETS (E26 avian leukemia oncogene) family is a diverse group of transcription factors involved in signal transduction, cell cycle progression, differentiation, angiogenesis, and invasiveness [15]. ETS proteins are mitogen-activated protein kinase (MAPK)–dependent transcription factors. They contain a conserved winged helix-turn-helix DNA-binding domain and regulate gene expression by binding to ETS-binding sequences in the promoter/enhancer regions of their target genes. These domains specifically recognize the 5′-GGAA/T-3′ sequence [16]. More than 27 ETS proteins have been identified in humans [17]. The role of ETS-1 and ETS-2 has been studied for many cancers. The Ras/Raf/MERK/Erk pathway is one of several downstream signaling cascades activated by the G protein-coupled Ras proteins. Once activated, an Erk kinase at the bottom of this cascade phosphorylates cytoplasmic substrates and may be translocated to the nucleus. In the nucleus, it phosphorylates transcription factors, some of which initiate the immediate and delayed early gene responses. Erk also phosphorylates several transcription factors including ETS, Elk-1, and SAP-1. In some cancers, signaling pathways downstream from Raf may be strongly activated in the absence of direct Ras involvement. Thus, in 60%-70% of melanomas, a closely related functional analog of Raf, B-Raf, is found in a mutated constitutively activated form. It remains unclear why proliferation in these melanomas is driven by mutant B-Raf rather than mutant Ras. Highly conserved ETS protein orthologs are present in several species including mouse, chicken, nematode, *Xenopus*, and *Drosophila*. We focused our study on two ETS genes, *Ets-1* and *Ets-2*. These genes seem to be derived from duplication of an ancestral gene that also gave rise to the Drosophila gene, *pointed* (*Pnt2*) [18,19]. *Pnt2* is involved in the differentiation of photoreceptor R7. Based on this known role of *ETS-1* and *ETS-2* in photoreceptor differentiation and the current lack of knowledge concerning the role of these transcription factors in normal murine retina, we decided to study the production and roles of these two proteins in the normal mouse retina including RPE and in the Tyrp1-TAg transgenic mouse model of pigmented ocular neoplasm. ETS-1 and ETS-2 are produced in various tissues [20]. The role of ETS-1 in cancer has been studied extensively [21]. However, much less is known about the role of this protein in the normal and pathologic central nervous system of which both the RPE and the neural retina are major components. The production of this protein may play a major role in the pathogenesis and may be predictive of aggressive cutaneous melanoma as it is present in melanocytic lesions [22]. It is also produced in various solid tumors including epithelial tumors, sarcomas, and astrocytomas [21]. High ETS-1 levels in breast, ovary, and cervical carcinomas are associated with a poor prognosis [23,24]. ETS-1 is a prognostic marker of breast cancer, independent of other tumor markers such as nodal status, tumor size, histological grade, or estrogen receptor status [25]. The presence of ETS-1 is associated with a high incidence of lymph node metastasis in the lung, colorectal, and squamous cell carcinoma [26,27]. ETS-1 is also present in large amounts in leukemic T cells [28]. The ETS-1 transcription factor is involved in two other major carcinogenic processes, metastasis and angiogenesis. The gene encoding this factor is coexpressed with the genes encoding uPA (urokinase type plasminogen activator) and MMP-1 (matrix metalloproteinase-1) in various types of tumor [29,30]. ETS-1 is also produced together with MMP-2 and MMP-9 in pancreatic cancer [31]. The importance of ETS-1 in cancers may be partly accounted for by the role of this factor in angiogenesis. Several members of the ETS family have a combinatorial effect on vasculature development [32]. Indeed, oligonucleotides or transdominant mutant ETS-1 molecules with dominant negative effects inhibit angiogenesis [33,34], consistent with a critical role for ETS-1 in angiogenesis. However, *ETS-1* null mice have no detectable vascular defects [35-37]. ETS-1 regulates several downstream effectors of angiotensin II including p21CIP, plasminogen activator inhibitor-1 (PAI-1), vascular cell adhesion molecule 1 (VCAM-1), and monocyte chemoattractant protein-1 (MCP-1) and plays a very important role in inflammation and vascular remodeling in response to angiotensine 2 (Ang II) [38] as shown by in vitro and in vivo experiments. This model makes it possible to determine whether this is also the case in a mouse model of eye cancer in which angiogenesis probably plays a major role in the development of the primary tumor and its local and distal propagation, leading to the formation of metastases.

ETS-2 has mostly been studied in association with Down syndrome. ETS-2 transactivates the *β APP* gene promoter [39] and the upregulation of this gene induces neuronal apoptosis [40,41]. However, ETS-2 has also been implicated in prostate cancer [42] and together with other factors including ETS-1, SRC-1 (v-src avian sarcoma [Schmidt-Ruppin A-2] viral oncogene homolog), AIB-1 (nuclear receptor coactivator 3) and NcoR (nuclear receptor co-repressor) [43,44], breast cancer. ETS-2 and ERM (ets variant 5) also significantly increase transcription of the gene encoding intercellular adhesion molecule-1 (ICAM-1) [45], which has a major role in uveal tumor growth [46]. The roles of these factors in the eye are unknown.

Here, we describe major roles for these transcription factors in a mouse model of ocular cancer. This model has been used as an ocular cancer mouse model to test new potential therapies for human choroidal melanoma [47]. Our study is the first to demonstrate the production of ETS-1 and ETS-2 in normal, whole mouse eyes during postnatal development and adulthood. Both ETS-1 and ETS-2 were detected in various ocular cell types. We also investigated the levels and roles of these factors in the mouse Tyrp-1-TAg transgenic model of ocular cancer. Levels of mRNA and protein for these two transcription factors were higher in abnormal mouse eyes during the development of tumors than in normal control eyes of the same age. We also demonstrated an upregulation of various known targets of these transcription factors that is part of a developmental pathway potentially involved in ocular cancer progression.

## Methods

### Animals

All animals were handled in compliance with the Association for Research in Vision and Ophthalmology (ARVO) statement for use of animals in ophthalmic and vision research. Animals were kept at 21 °C with a 12 h light (100 lx)/12 h dark cycle and with free access to food. We studied normal CB6 mice (WT) and transgenic CB6 Tyrp-1-TAg mice [10] between the ages of P15 and three months.

### Riboprobe synthesis

The *ETS-1* and *ETS-2* riboprobes were 451 and 503 bp long, respectively, and were synthesized using a polymerase chain reaction (PCR)-based in situ hybridization technique as previously described [48,49]. PCR was performed with *ETS-1* or *ETS-2* gene-specific primers, incorporating a binding site for T7 RNA polymerase. Purified PCR products were then used for transcription reactions with T7 forward and reverse primers.

### In situ hybridization

*ETS-1* and *ETS-2* riboprobes were labeled with a 10X digoxigenine (DIG) RNA labeling kit (Promega, Charbonnieres, France). In situ hybridization was performed on deparaffinized, rehydrated 5 µm eye sections from CB6 control animals. Tissue sections were incubated overnight at 65 °C with the probes and washed with 1X Stringent Wash Concentrate (Dako, Glostrup, Denmark) according to the manufacturer’s instructions. Tissue sections were then incubated for 1 h at room temperature with anti-DIG–AP (alkaline phosphatase-conjugated antibody against DIG) and rinsed in PBS. Tissue sections were incubated with the AP substrate, nitro-blue tetrazolium/5-bromo-4-chloro-3-indolyl-phosphate (NBT/BCIP), for 30 min in the dark. Hybridized tissue sections were examined under a light microscope (LEICA, Solms, Germany). Similar amounts of probe (sense or antisense) were applied to each slide, and all slides were treated similarly in the same experiment to ensure that they could be compared. Experiments were performed in triplicate, and results were analyzed by two independent investigators.

### Peroxidase/DAB immunohistochemistry

Deparaffinized, rehydrated 5 µm eye and brain sections from CB6 control mice were incubated overnight at 4 °C with antibodies against ETS-1 (1:500; sc-350, Santa Cruz Biotechnology, Santa Cruz, CA) or ETS-2 (1:500; sc-351, Santa Cruz Biotechnology) diluted in Dako antibody diluent. Bound antibodies were detected with the ChemMate peroxidase/DAB rabbit/mouse detection kit (Dako) according to the manufacturer's instructions.

### Immunohistofluorescence

Deparaffinized, rehydrated 5 µm eye sections were incubated overnight at 4 °C with a 1/500 dilution of antibody against ETS-1 (sc-350; Santa Cruz Biotechnology) or ETS-2 (sc-351; Santa Cruz Biotechnology) in blocking solution. Sections were washed in 1X PBS and incubated with a 1/200 dilution of goat anti-rabbit Alexa Fluor 488 antibody in a dark chamber. Tissue sections were then washed with PBS in the dark and mounted in DakoCytomation fluorescence mounting medium. Tissue sections were stored at 4 °C until microscopic analysis.

### Reverse-transcription polymerase chain reaction

CB6 Tyrp-1 (n=5) and control mouse (n=5) eyes were removed at postnatal (P) stages P15, P20, P25, and P30 and at three months (adult). Total RNA was extracted with an extraction reagent (TRIzol; Invitrogen-Gibco, Paisley, UK) according to the manufacturer’s instructions. Total RNA (1 µg) was reverse-transcribed with reverse transcriptase (SuperScript II; Invitrogen-Gibco) and oligo-dT primer according to the manufacturer’s instructions. For semi-quantitative PCR, the number of cycles, amount of cDNA, and annealing temperature were optimized (data not shown). The cyclophilin gene was amplified as an internal control. PCR was then conducted in 10 µl of reaction mixture containing 0.5 µl cDNA, 1 µl 10X PCR buffer (Promega, Madison, WI), 1 µg of each specific (5’-3’) and (3’-5’) primers corresponding to each cDNA of interest amplified by PCR, 0.5 µg of each cyclophilin primer (5’-3’ and 3’-5’), 0.2 mM dNTP, 1.5 mM MgCl_2_, and 0.1 U Taq DNA polymerase. An initial denaturation step at 94 °C for 2 min was followed by 29 cycles of heating for 1 min at 94 °C, 1 min at 55 °C, and 1 min at 72 °C.

The *ETS-2* primers (5′-CAT CCT CTG GGA ACA TCT AG-3′ and 5′-TAC CCG CTG TAC ATC CAG TA-3′) amplified a 451 bp product. The *ETS-1* primers (5′-AAA GAG TGC TTC CTC GAG CT-3′ and 5′-AGG CTG TTG AAG GAT GAC TG-3′) amplified a 503 bp product. The cyclophilin primers (5′-TGG TCA ACC CCA CCG TGT TCT TCG-3′ and 5′-TCC AGC ATT TGC CAT GGA CAA GA-3′) amplified a 311 bp product. We also used *GAPDH* as a second control gene (data not shown).

Signal was quantified with Scion image software (Frederick, MD). The experiments were performed three times. We also tested different groups of primers for each gene.

### Statistical analysis

All results are expressed as the mean±SD. The results were compared using analysis of variance (ANOVA) and Student’s *t*-test. A p<0.001 was considered statistically significant.

### Western blotting

Total proteins were extracted from CB6 control (n=5) and CB6 Tyrp-1 mouse (n=5) eyes at P15, P20, P25, P30, and three months of age using an extraction reagent (TRIzol; Invitrogen-Gibco) according to the manufacturer’s instructions. Proteins (50 μg; Bradford protein assay) were separated by electrophoresis (SDS–PAGE in a 10% polyacrylamide gel). Separated proteins were transferred onto a nitrocellulose membrane (Trans-Blot Transfer Medium; Bio-Rad, Hercules, CA), which was then blocked by incubation for 1 h with 5% nonfat milk. Membranes were then incubated overnight with rabbit anti-ETS-1 antibody (sc-350; Santa Cruz Biotechnology), anti-ETS-2 antibody (sc-351; Santa Cruz Biotechnology), or a goat anti-β-actin antibody (Santa Cruz Biotechnology). They were then washed and incubated for 1 h with a horseradish peroxidase-linked anti-rabbit or anti-goat secondary antibody (Santa Cruz). Proteins were detected by enhanced chemiluminescence (ECL; PerkinElmer Life and Analytical Sciences Inc, Courtaboeuf, France). The experiments were performed three times.

## Results

### *ETS* mRNA and protein localization in adult mouse eyes

**Figure 1:**
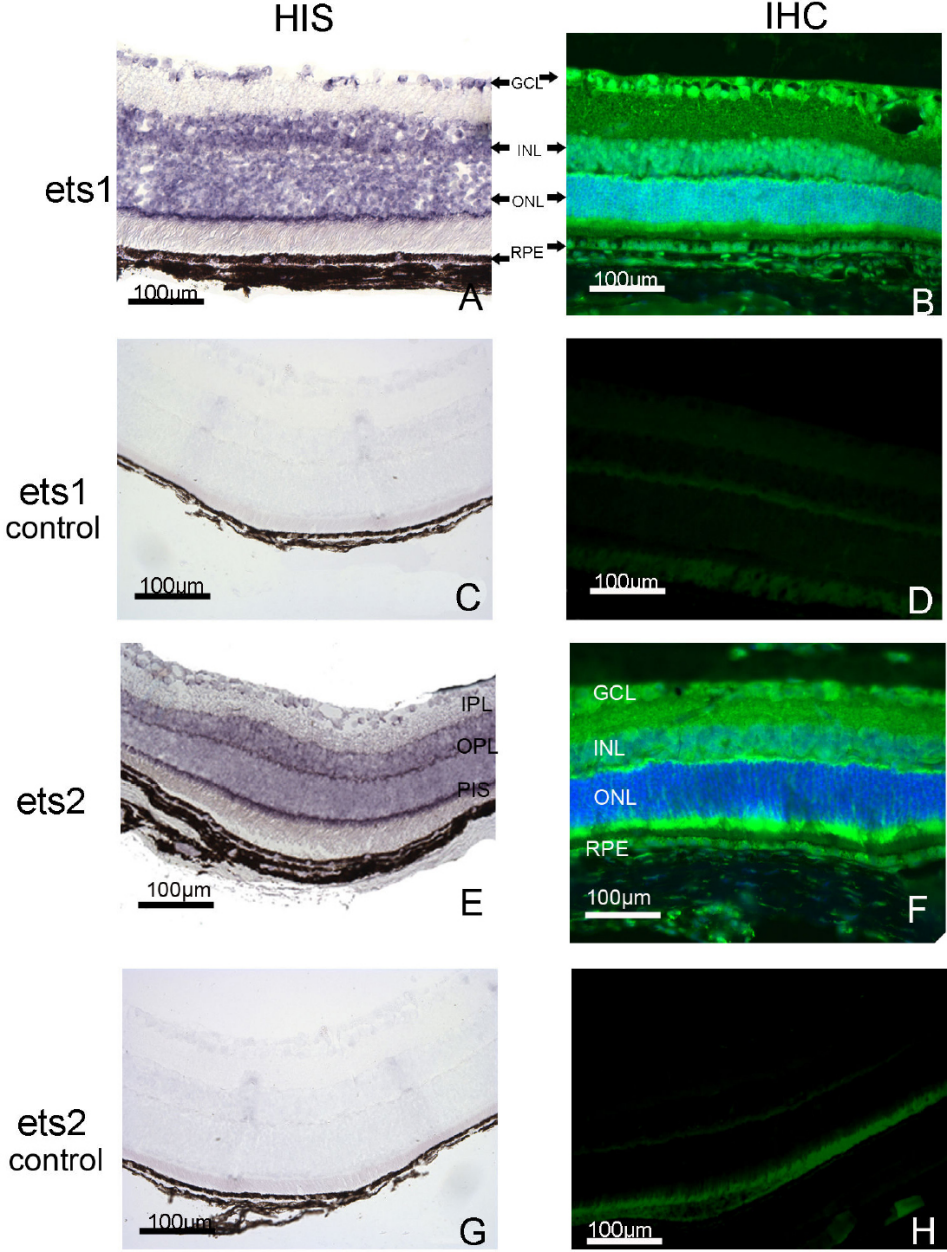
Distribution of *ETS-1* and *ETS-2* mRNAs and proteins in the adult mouse eye. The *ETS-1* and *ETS-2* mRNAs were detected in adult mouse tissue sections by in situ hybridization (HIS), and their distributions are shown (**A** and **E**, respectively). The ETS-1 and ETS-2 proteins were detected by immunohistofluorescence (IHC) in adult mouse tissue sections. The specific immunostaining patterns obtained are also shown (**B** and **F**, respectively). The retinal tissue distributions of ETS-1 (**B**) and ETS-2 (**F**) proteins appeared to be somewhat different whereas the distribution of the transcripts corresponding to these proteins (**A** and **E**) seemed to be similar in adult mouse retina. The *ETS-1* (**C**)and *ETS-2* (**G**) mRNAs show the negative control in adult mouse tissue sections by in situ hybridization obtained with sense riboprobes. The ETS-1 (**D**) and ETS-2 (**H**) immunohistofluorescence shows the negative control in adult mouse tissue section. The results were obtained with the second antibody alone. The nucleus was counterstained with DAPI. The scale bar represents 100 µm. GCL: ganglion cell layer; IPL: inner plexiform layer; INL: inner nuclear layer; OPL: outer plexiform layer; ONL: outer nuclear layer, RPE: retinal pigment epithelium; PIS: photoreceptor inner segment.

The genes, *Ets-1* and *Ets-2*, seem to be derived from duplication of an ancestral gene that also gave rise to the Drosophila gene, *pointed* (*Pnt2*). This gene is involved in the differentiation of the photoreceptor, R7, suggesting that ETS-1 and ETS-2 may be involved in the development and/or biological functions of the neural retina. We used in situ hybridization to study the distribution of *ETS-1* and *ETS-2* mRNA in paraffin-embedded sections of adult mouse eye tissue. We detected mRNA for both *ETS-1* and *ETS-2* in the neuroretina (Figure 1A,E). We found mRNA for these factors in the ganglion cell layer (GC), inner nuclear layer (INL), outer nuclear layer (ONL), and photoreceptor inner segments (PIS) but not in the photoreceptor outer segments. Both transcripts were also present in the adult retinal pigment epithelium (RPE; Figure 1A,E) and uveal melanocytes. *ETS-1* mRNA was not produced in detectable amounts in the outer plexiform layer whereas *ETS-2* mRNA was clearly produced in significant amounts in this layer, which consists primarily of fibers and synapses. This observation was validated in several sets of repeated experiments.

**Figure 2:**
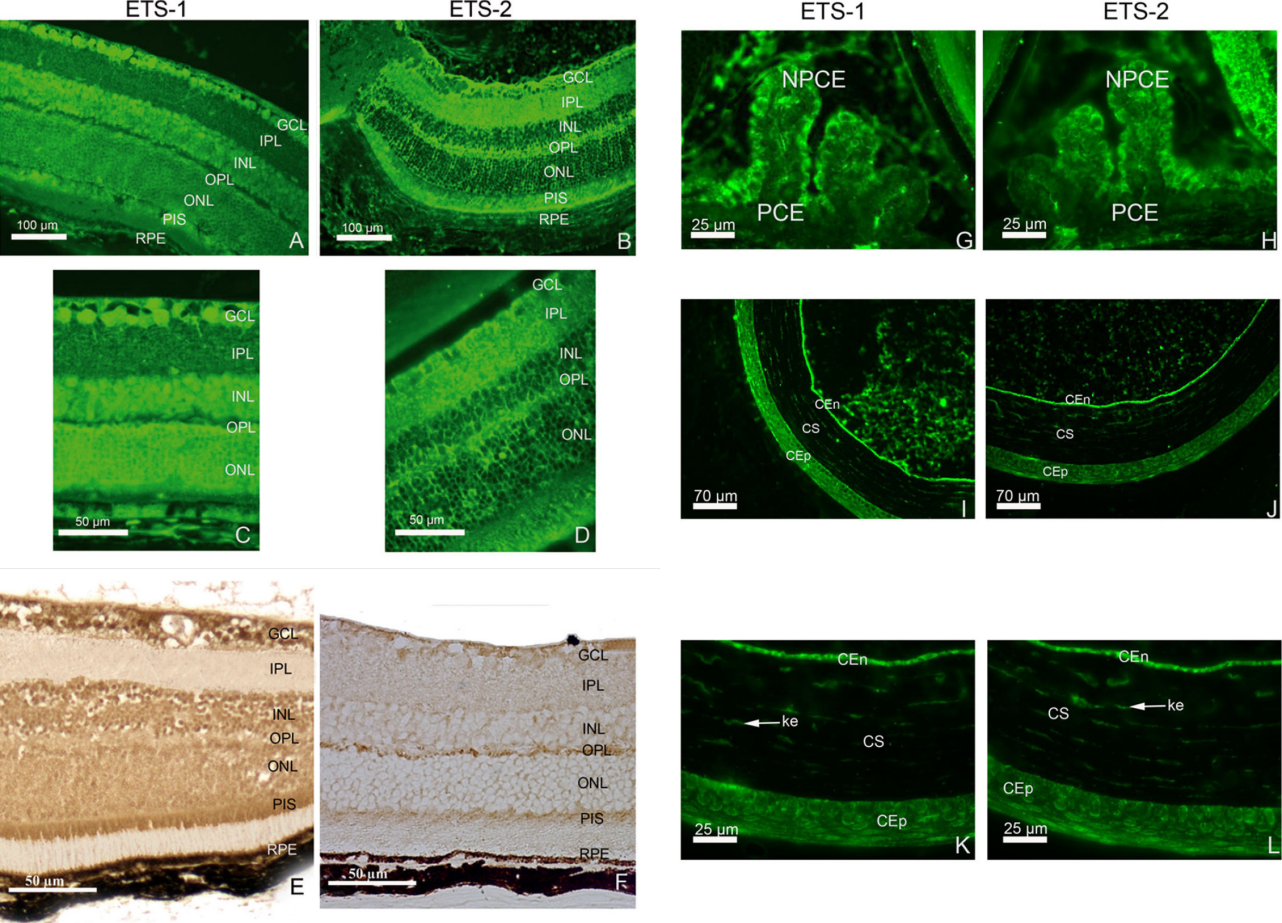
Distribution of ETS transcription factor proteins in mouse adult retina, cornea, and ciliary body as determined by immunohistofluorescence. Immunohistofluorescence for ETS-1 and ETS-2 is shown (**A**-**D)**. ETS-1 (**A** and **C**) is present in the ganglion cell layer (GCL) and inner nuclear layer (INL) with intense homogeneous staining in the outer nuclear layer (ONL). It is also detected in the photoreceptor inner segment (PIS) and retinal pigment epithelium (RPE). ETS-2 (**B** and **D**) is also present in significant amounts in the GCL. Weak ETS-2 immunoreactivity is observed in the INL and ONL. In these layers, at higher magnification, very few cell bodies are immunostained. The immunolabeling detected seems to be restricted to the nuclear membranes whereas the nucleoplasm seems to be devoid of ETS-2 immunolabeling. In contrast to what was observed for the retinal distribution of ETS-1 immunoreactivity, strong ETS-2 immunolabeling is observed in the inner plexiform layer (IPL) and outer plexiform layer (OPL). As observed for ETS-1, the PIS and RPE display marked ETS-2 immunostaining, although the RPE seems to be less strongly stained for ETS-2 than for ETS-1. ETS-1 (**E**) and ETS-2 (**F**) protein distributions are shown in the adult retina with the DAB immunostaining protocol. We observed ETS-1 (**G**) and ETS-2 (**H**) immunolabeling in the adult ciliary body. In the ciliary body, ETS-1 and ETS-2 proteins are detected in the nonpigmented ciliary epithelial cells (NPCE). No significant labeling is observed in the pigmented ciliary epithelial cells (PCE). ETS-1 immunolocalisation was detected in the adult cornea at low (**I**) and high (**K**) magnification. ETS-2 immunolocalisation was detected in the adult cornea at low (**J**) and high (**L**) magnification. In the cornea, ETS-1 and ETS-2 immunoreactivities are observed in the epithelium (CEp), the stroma (CS), the stromal keratocytes (ke, arrows), and the endothelium (CEn).

We also performed immunohistofluorescence and immunohistochemistry analyses to determine the cellular distribution of ETS-1 and ETS-2 immunoreactivity in the adult mouse retina (Figure 1B,F and Figure 2). The negative controls of ETS-1 (Figure 1C) and ETS-2 (Figure 1G) in situ hybridization and the negative controls of ETS-1 (Figure 1D) and ETS-2 (Figure 1H) immunohistofluorescence are presented in Figure 1. The retinal pattern of immunoreactivity for ETS-1 matched the retinal distribution of *ETS-1* transcripts (Figure 1B). We detected ETS-1 immunoreactivity in the nerve fiber layer (NFL), the ganglion cell layer (GCL), the INL (with apparent stronger labeling of the bipolar neuron cell bodies on either side of the INL and significant labeling probably of the cell bodies of amacrine cells). Horizontal cell bodies were detected at the interface of the ONL and INL on the basis of morphological criteria. However, double-labeling experiments are required to confirm unambiguously the nature of the cell bodies immunolabeled for ETS-1 in the INL and in the region close to the ONL. No significant ETS-1 immunostaining was observed in the IPL whereas ETS-2 immunostaining was strong in this cell layer in adult mice. ETS-1 immunoreactivity in the OPL was detectable but much weaker than ETS-2 immunoreactivity in the same layer, which was very strong (Figure 1F). ETS-1 immunolabeling in the ONL was weaker than that in the INL, but both the nuclei and cytoplasm of the ONL cells appeared to be stained. The RPE displayed strong levels of immunoreactivity for ETS-1 (Figure 2A,C), and significant ETS-1 immunostaining was also detected in uveal melanocytes. ETS-2 immunoreactivity was detected in the adult mouse retina, but its cellular distribution differed from that of ETS-1 (Figure 1D). The ETS-2 antibody used seemed to almost exclusively stain the cytoplasm surrounding the nuclei of cells in the GCL, ONL, INL, and RPE cell layer. We also detected distinct ETS-2 immunostaining in the nerve cell layer, GCL, IPL, and PIS layer (Figure 2B,D,F). We found positive immunolabeling for ETS-2 in the nucleus and cytoplasm of some cells in the INL, but most cell bodies of the INL displayed essentially cytosolic ETS-2 immunostaining mostly at the periphery of the labeled cells. In some cases, the staining surrounded the nuclei and appeared to be very close to, if not associated with, the cytoplasmic membranes. Examination of eye tissue sections under a fluorescence microscope at low and medium magnification did not reveal significant immunoreactivity signals for ETS-2 in the ONL. However, at high magnification, the nuclei of ONL cells resembled ghost nuclei surrounded by the weakly labeled ETS-2-immunoreactive cytosol. Immunoperoxidase labeling for ETS-2 was positive in all the cell bodies located in the GCL, INL, ONL, and RPE cell layer, demonstrating greater homogeneity. With this technique, only the cytosol appeared to be significantly labeled, although much less strongly compared to ETS-1 immunostaining. Significant ETS-2 immunostaining of the OPL was clearly visible. The ETS-2 immunoreactivity in the RPE and uveal melanocytes was significant but much weaker than ETS-1 immunoreactivity in the same cell types. We cannot exclude the possibility that Müller cells were also immunostained for ETS-2 whereas this seems less likely for ETS-1. No difference in the respective patterns of ETS-1 and ETS-2 immunostaining was detected during postnatal ocular development between P15 and adulthood. The most striking differences between the retinal immunostaining patterns for ETS-1 and ETS-2 were the stronger immunolabeling of the IPL and OPL for ETS-2 than for ETS-1 and the much lower immunoreactivity of the ONL for ETS-2 than for ETS-1.

We also detected immunoreactivity for ETS-1 and ETS-2 in other adult mouse eye structures (Figure 2) such as the ciliary bodies, corneal epithelium, keratocytes, and corneal endothelial cells. In the mouse adult retina, ETS-1 is mostly present in the nuclei while ETS-2 is mostly present in the cytosol with smaller amounts present in the nuclei. ETS-2 was present in large amounts in dendritic, synaptic, and axonal retinal areas. The possibility of a structural role for ETS-2 in addition to its role as a transcription factor cannot be ruled out for retinal neurons. These findings suggest that ETS-1 and ETS-2 have overlapping but distinct roles in the biological functions of the eye.

### *ETS-1* and *ETS-2* are upregulated in a murine model of ocular cancer

**Figure 3:**
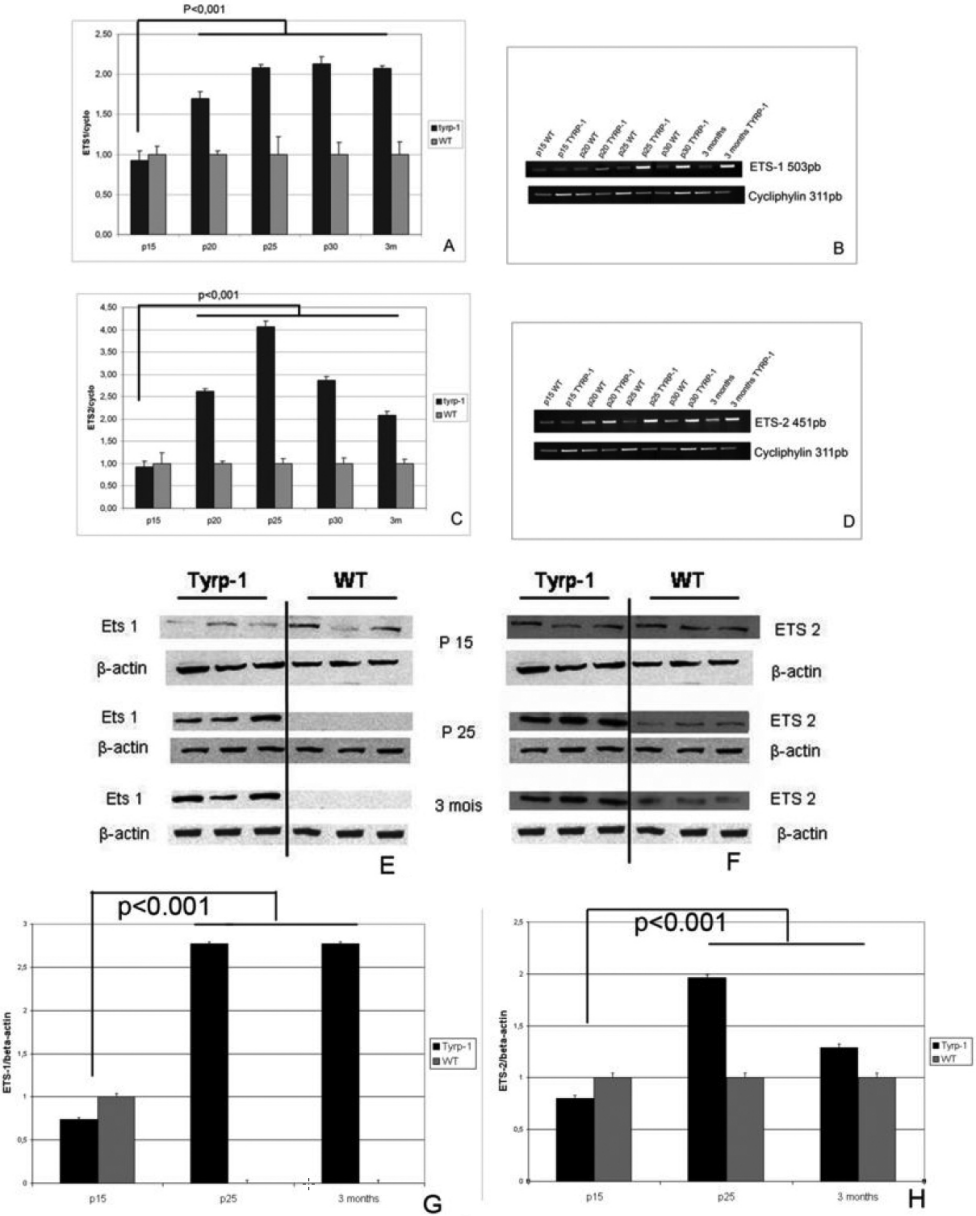
Overproduction of *ETS-1* and *ETS-2* mRNAs and proteins in a mouse model of ocular neoplasm (Tyrp-1), with respect to control mice (WT) between P15 and three months. mRNA levels were determined by semi-quantitative RT–PCR. The values shown correspond to the levels of overproduction of ETS-1 and ETS-2 with respect to levels in normal control mice at the same age. The graphs (**A** and **C**) show the intensity level of each PCR band, corresponding to ETS-1 (**A**) and ETS-2 (**C**) as measured by densitometric analysis. The cyclophilin is used as an internal standard control. The PCR bands for ETS-1 (**B**) and ETS-2 (**D**) are shown. The relative levels are calculated as the ratio of intensities of each ETS band to the cyclophilin band for each lane. Error bars indicate SEM. Proteins were detected by western blotting (**E** and **F**). Levels were quantified using Scion image and normalized using β-actin as an internal standard (**G** and **H**).

We investigated the roles of ETS-1 and ETS-2 in the development of pigment neoplasms using semi-quantitative RT–PCR to compare mRNA levels for *ETS-1* and *ETS-2* in normal (WT) and tumoral mouse model (TYRP-1-TAg) whole eyes. Cytophilin was used as an internal standard for relative quantification of *ETS-1* and *ETS-2* mRNA levels. We determined *ETS-1* and *ETS-2* mRNA levels at P15, P20, P25, P30, and three months of age (Figure 3A,C). The *ETS-1*/cyclophilin mRNA ratio in whole eyes almost doubled between P25 and three months in TYRP-1-TAg mice but not in WT mice (Figure 3A). *ETS-2* mRNA levels were significantly higher in the TYRP-1-TAg model than in wild-type mice. At P25, *ETS-2* mRNA levels were more than four times higher in TYRP-1-TAg than in the wild-type. *ETS-2* mRNA levels were slightly lower in TYRP-1-TAg than in WT mouse eyes at P30 and at three months (Figure 3C).

We investigated the consequences of this upregulation of *ETS-1* and *ETS-2* mRNA levels by western blotting to compare protein levels. We studied ETS-1 and ETS-2 protein levels at the same time points in Tyrp1-TAg and WT mice (Figure 3E-H). ETS-1 and ETS-2 protein levels were significantly higher in Tyrp1-TAg than in wild-type mice for all time points between P25 and three months. ETS-1 protein was first detected in WT eyes at P25 by western blotting. The exposure time allowing the readily detection of ETS-1 and ETS-2 by western blotting in the retina of wild-type CB6 mice gave highly saturated signals for ETS-1 protein extracted from eyes affected by ocular tumor. However, the levels of ETS-2 protein in eyes with ocular tumor could still be quantified and interpreted using this exposure time. The ETS-1 signal was not detected on western blots of normal eyes using shorter exposure times but was readily detected and quantified on western blots of ocular tumors from transgenic mice. These results show a marked increase in protein levels for ETS-1 and ETS-2 after P25 in transgenic mice compared to the control mice.

Our findings suggest that the genes encoding ETS-1 and/or ETS-2 may play a role in the emergence and/or progression of ocular tumor.

### Expression of ETS-1 and ETS-2 in the mass of tumor cells

**Figure 4:**
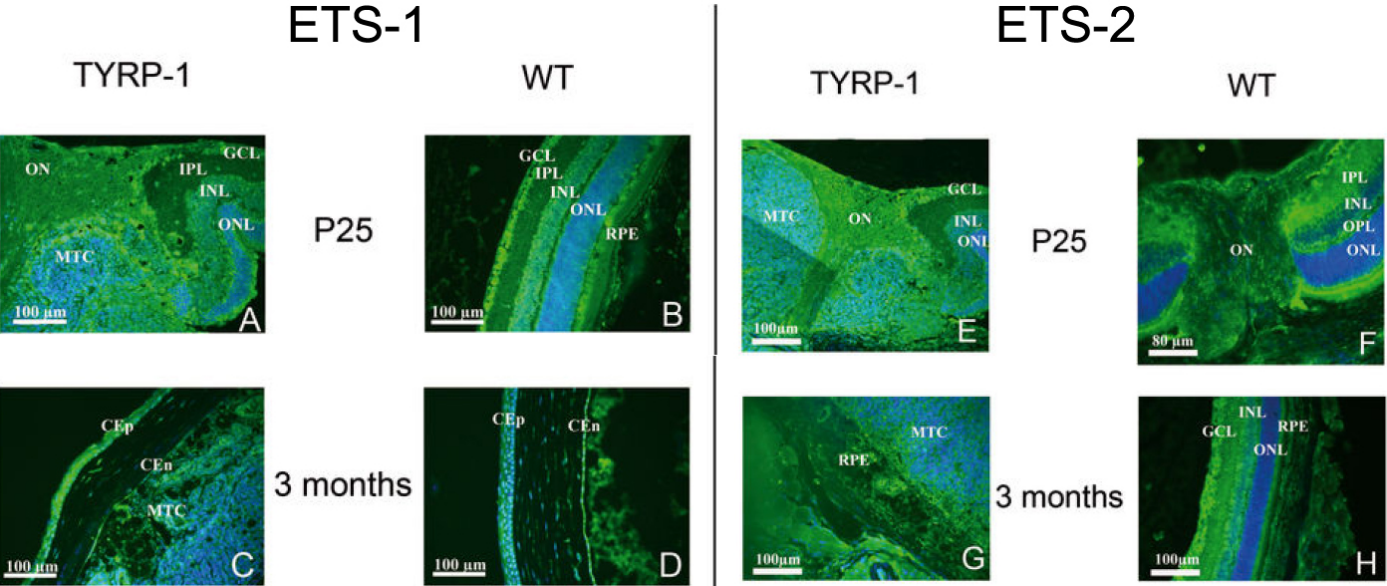
ETS-1 and ETS-2 distribution in the tumor. We detected ETS-1 protein in the tumor by immunohistofluorescence on 5 µm paraffin-embedded sections of whole mouse eyes. Distributions of ETS-1 in the posterior pole of Tyrp-1 transgenic mouse eye tissue sections (**A**) and in tissue sections from control mice (**B**) are shown at P25; ETS-1 is significantly expressed in the mass of tumor cells. At three months, the melanoma tumor reaches the cornea of Tyrp-1 transgenic mice eyes (**C**) whereas in control mice, only lens fibers can be observed (**D**). ETS-1 levels are expressed within the mass of tumor cells (MTC). We detected ETS-2 protein by immunofluorescence on 5 µm paraffin embedded tissue sections. Distributions of ETS-2 protein at P25 in the posterior pole of Tyrp-1 (**E**) and control mice (**F**) are also shown; ETS-2 is significantly expressed in the mass of tumor cells. Distribution of ETS-2 protein at three months in Tyrp-1 (**G**) and control mice (**H**). ETS-2 is present within all cells contained within the mass of tumor cells (MTC). The nucleus was counterstained with DAPI. The scale bar represents 100 µm.

We previously observed ETS-1 and ETS-2 overproduction. However, the results obtained did not determine whether ETS-1 or ETS-2 was increased exclusively in the retina and/or RPE per se and in the ocular tumor only or throughout the whole eye. We addressed this issue using immunohistofluorescence to detect these two proteins in the murine ocular tumor (Figure 4). At P15 and P20, the amounts and distribution of these proteins were similar between Tyrp-1 TAg and wild-type mice.

We observed specific cellular immunostaining for ETS-1 in the retinal site surrounding the optic nerve at the point of tumor development at P25 (Figure 4A). The neuroretina was completely disorganized at the posterior pole of transgenic mice (Figure 4B) with a mass of cells producing ETS-1. This mass of tumor cells (MTC) forms a “collar button” or “mushroom”-like structure, which is highly characteristic of choroidal melanoma. At three months, this disorganization was more extensive, spreading from the posterior pole to the anterior. We observed a structure corresponding to a thickening of the pigmentary epithelium producing ETS-1 (Figure 4C) both in malignant RPE cells and in malignant melanocytes. Like ETS-1, ETS-2 was produced in the MTC at the posterior pole at P25 at higher levels in the transgenic mice than in control (WT) mice (Figure 4E,F). At three months, we also observed a thickening of the pigmentary epithelium, producing ETS-2 protein at higher levels than in WT mice (Figure 4G,H).

### Overexpression of ETS-1 and ETS-2 target genes in ocular cancer

We previously showed that the transcription factors, ETS-1 and ETS-2, were both upregulated from P20 to three months in our ocular cancer model. We therefore assessed whether this overproduction of ETS-1 was correlated with an upregulation of some of its known target genes in our model of ocular cancer. Three genes are known to be regulated by ETS-1, *MCP-1* (monocyte chemoattractant protein-1), which has growth promoting effects (reviewed in [50]); *p16INK4A* cyclin-dependent kinase, which is directly activated by ETS-1 and involved in replicative senescence; and *PAI-1* (plasminogen activator inhibitor type-1), which is considered a major regulator of tumor invasion and metastasis and of cancer-related angiogenesis [51]. *ICAM-1*, which is involved in angiogenesis, is directly controlled by ETS-2 [45].

**Figure 5:**
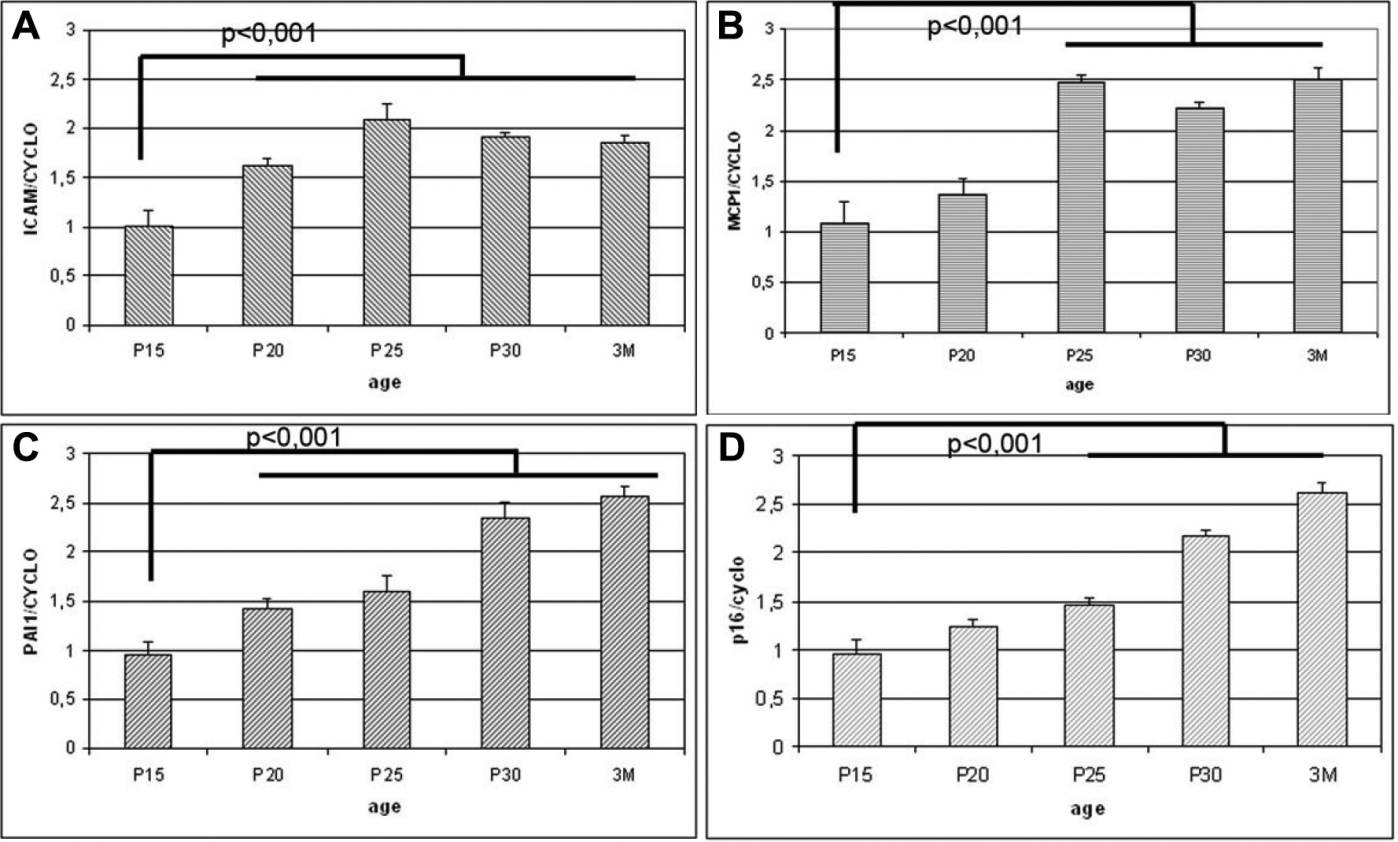
Expression of several known target genes of ETS-1 and ETS-2. *ICAM-1*, *MCP-1*, *PAI-1*, and *p16* mRNA levels were evaluated by semi-quantitative RT–PCR and normalized using cyclophilin mRNA as a control. These mRNA levels were compared with those in control mice of the same age. In **A**, we observed that ICAM-1 is upregulated in Tyrp-1 model from p20 to 3 months. In **B**, we observed that MCP-1 is upregulated in Tyrp-1 model from p25 to 3 months. In **C**, we observed that PAI-1 is upregulated in Tyrp-1 model from p20 to 3 months. In **D**, we observed that p16 is upregulated in Tyrp-1 model from p25 to 3 months.

We performed semi-quantitative RT–PCR assays on total RNA from the eyes of normal mice (WT) and transgenic mice (Tyrp-1-TAg). We quantified mRNA levels for these target genes using the endogenous cyclophilin gene for normalization. Levels of mRNA were significantly higher for all these target genes in Tyrp-1-TAg mice than in wild-type mice from P20 to three months (Figure 5). This result is consistent with the higher *ETS-1*/*ETS-2* mRNA and protein levels observed in these mice. Our data suggest that ETS-1 and ETS-2 are involved in one or more signaling pathways activating the expression of a set of genes involved in ocular tumor progression and the probable acquisition of high metastatic potential by this tumor. These findings demonstrate the relevance of one or more signaling pathways, that are downstream ETS-1 and ETS-2, and the relevance of crucial molecular building blocks of one or more of these pathways, in the ocular tumor development occurring in the transgenic mouse model studied.

## Discussion

We describe here the expression profiles for ETS-1 and ETS-2, two members of the ETS family of transcription factors. We first investigated the pattern of *ETS-1* and *ETS-2* gene expression in mouse eyes. Previous studies have reported the detection of ETS transcription factors including PEA3 [52], ELF3 [53] and ESE-1 [54] in rodent and human retinas. In *Drosophila,* *Pnt2*, the ortholog of *ETS-1* and *ETS-2*, is involved in eye development [55-57]. Our study is the first to demonstrate the presence of ETS-1 and ETS-2 in the mouse eye and to show that these two transcription factors have different spatial distributions in the mouse neuroretina. We detected ETS-1 immunoreactivity in the ganglion cell layer, the inner and outer nuclear layers, and the photoreceptors inner segments. No significant ETS-1 immunoreactivity could be detected in any of the plexiform layers. We also detected ETS-1 immunostaining in the RPE. ETS-2 immunoreactivity was detected in the GCL, the INL, the PIS, and the RPE, but no significant ETS-2 immunolabeling could be detected in the ONL. This absence of ETS-2 immunostaining in the ONL suggests that ETS-2 is not directly involved in specific photoreceptor functions. Although no ETS-2 immunostaining could be detected in the ONL, significant ETS-2 immunoreactivity could be observed in the OPL. These findings suggest that ETS-2 might be involved in synaptic transmission. Further studies are required to define the role of ETS-1 and ETS-2 in the different retinal layers. A major finding of our study is the striking difference observed between the cellular immunostaining pattern observed for ETS-1 and that observed for ETS-2. While ETS-1 appeared to be localized mostly in retinal cell nuclei, ETS-2 seemed to be mostly in the cytosol of retinal cells. Electron microscopy and live imaging experiments are required to unambiguously confirm these observations. Our observations in the retina are consistent with those previously reported for other parts of the central nervous system (CNS; data not shown). The differences in tissue and intracellular ETS-1 and ETS-2 retinal immunostaining patterns suggest that ETS-1 and ETS-2 might have different biological functions in the adult retina [58]. This contrasts with previous general studies of the distribution of ETS-1 and ETS-2. Indeed, the chicken ETS protein, which contains both the ETS-1 and ETS-2 domains, is uniformly distributed between the cytoplasm and nucleus whereas in human and other mammalian tissues producing these transcription factors, ETS-1 localization is generally cytoplasmic and ETS-2 localization is nuclear. Differences in immunostaining patterns for these two proteins in neural tissues are consistent with the notion of different biological functions for ETS-1 and ETS-2 in the CNS [59].

We then investigated *ETS-1* and *ETS-2* gene expressions in a mouse model of pigmented ocular neoplasm. We showed that *ETS-1* and *ETS-2* mRNA and protein levels were higher in these mice than in wild-type mice. This is consistent with previous studies [17,60,61] that demonstrated the fact both ETS-1 and ETS-2 may play important roles in the development of ocular cancer. We also investigated the possible role of another member of the ETS family in the development of this tumor. GA BINDING PROTEIN (GABP) is an ETS transcription factor required for normal cell cycle progression [62]. We used semi-quantitative RT–PCR to assess differences in GABP levels between Tyrp-1-TAg mice and controls. No significant difference was found between transgenic and control mice for *GAPB* mRNA levels or in the mRNA levels of GABP target genes, *Tymps*, *Pol-α*, and *Skp2* (data not shown). We have so far not found any evidence suggesting that the increased expression of ETS-1 and ETS-2 observed in these mouse ocular neoplasms resulted from the inactivation of p53 or Rb. The importance of the large T antigen interaction with Rb proteins and with p53 in SV40 transformation is well established. However, is this the whole story or do other T antigen activities contribute to the tumorigenic phenotype? Indeed, genetic studies suggest that the inactivation of pRb and p53 may not account for the full transformation potential of the T antigen [63,64]. Indeed, several cellular T antigen binding proteins have been identified that, based on their known functions, have potential to contribute to transformation and possibly progression of T antigen induced mouse tumors [6]. The involvement of chromatin and histone deacetylation in SV40 T antigen transcription regulation has been recently demonstrated [65]. Its consequences on *ETS-1* and *ETS-2* gene expression remain to be investigated. During malignant transformation, cancer cells acquire genetic mutations that override the normal mechanisms controlling cellular proliferation. Importantly, malignant progression has been shown to be triggered and/or accelerated by epigenetic mutations caused by alterations of DNA Methyltransferase-1 (*DNMT*) [66-69], histone acetyltransferase (*HAT*), Histone deacetylases (*HDAC*s) genes, and other mutator or modifier genes. Histone tail modifications along with DNA methylation are the most studied epigenetic events related to cancer progression [70]. Another area, which still remains to a large extent a terra incognita, is related to the transcription factors controlling ETS-1 and ETS-2 expression, although several ETS transcription factors have been shown to be downstream effectors of the Ras/Raf/MERK/Erk pathway. Our study demonstrates that ETS-1 and ETS-2 play a specific role in the development of T antigen induced RPE tumors.

Penna et al. [10] previously developed a transgenic mouse model in which the SV40 T antigen induces RPE tumor formation. This transgenic mouse model recapitulates many features of human choroidal melanoma. Indeed, the tumoral cells in this model develop an appearance similar to human choroidal melanoma cells including increased basophilia, nuclear and cytoplasmic polymorphism, prominent nucleoli, abundant mitosis with a tendency to metastasize, and expression of S100 and HMB-45 antigens. Furthermore, metastases in this model mostly develop in the liver, the major location for human choroidal melanoma metastasis. In the model we investigated, the major site of metastasis is the brain. It should be stressed that 5% of human choroidal melanomas have metastasis in the brain and not in the liver, particularly when the human choroidal melanoma occurs close to the optic disk. Therefore, upregulation of *ETS-1* and *ETS-2* could also occur in choroidal melanoma. This hypothesis has been confirmed by recent findings. Indeed, microarray gene expression profiling analysis by Harbour and Onken [71] (and personal communication) showed that *ETS-2* mRNA levels in human choroidal melanoma were four times higher than those in adult normal melanocytes. These findings are consistent with those of our study, indicating that ETS-2 is indeed increased in the Tyrp-1 TAg transgenic mouse ocular pigmented neoplasms and human choroidal melanoma. The results obtained highlight the clinical relevance of this transgenic mouse model for testing new drugs to potentially overcome the high level of chemical resistance of uveal melanomas [72,73].

Both ETS-1 and ETS-2 were produced at higher levels in Tyrp-1 TAg mice than in controls. Interestingly, in triplicate experiments using semi-quantitative PCR and western blotting to compare ocular tumors in Tyrp-1 mice with WT eyes at the same age, we found that *ETS-2* mRNA levels were higher than *ETS-1* mRNA levels, but ETS-1 protein levels were higher than ETS-2 protein levels. These results may be accounted for by different posttranscriptional regulatory mechanisms. Primary tumors and their various developmental stages can now be characterized molecularly by comparative whole genome expression profiling, the use of chips for mRNA detection, and proteomic techniques. MicroRNA expression in tumors was recently shown to provide valid specific signatures for each type of tumor [74,75]. The study of other types of regulatory RNAs might increase the accuracy of molecular characterization for each tumor. Different posttranscriptional regulation of *ETS-1* and *ETS-2* mRNAs by specific microRNAs and/or RNA-binding proteins could potentially explain our findings. Our observations could also be explained by epigenetic changes in tumor cells having differential effects on the regulation of genes encoding transcription factors and/or cotranscriptional regulators of *ETS-1* and *ETS-2*. Further experiments are required to test these hypotheses. For example, the role of Protein Kinase Cα (PKCα) should be explored because it is implicated in cell proliferation, cell migration, and tumor cell invasion in melanoma [76-79] and increases the stability of the ETS-1 protein [80].

One role for ETS-1 and ETS-2 in ocular cancer and choroidal melanoma may be mediated through their increased transcriptional activity and upregulated expression of their target genes involved in angiogenesis and/or metastatic propagation. ETS-1 and ETS-2 are activated by phosphorylation through Ras/mitogen-activated protein kinase signaling [81] but may also be repressed by serine phosphorylation [82,83]. Active ETS proteins can transactivate targeted genes. We studied the expression of target genes encoding ICAM-1, PAI-1, MCP-1, and p16 to determine the potential roles of ETS-1 and ETS-2 in the development of this tumor. We demonstrated by semi-quantitative RT–PCR that ETS-1 and ETS-2 target genes were upregulated from P20 to the age of three months in these mice, consistent with our observations for *ETS-1* and *ETS-2* mRNA and protein levels. These findings strongly suggest that both ETS-1 and ETS-2 are upregulated in this mouse model of ocular tumor with higher levels of transcriptional activity than in control mice. These effects may be involved in the pathogenic mechanisms of this disease. Most ETS factors are oncogenic [17], and the upregulation of *ETS* gene expression has been described in many types of human tumors. The levels of expression of these genes are correlated with invasion and metastasis and may be useful for predicting tumor progression in cancer patients. ETS-1 has also been implicated in various pathways involved in tumor angiogenesis through the activation of various target genes. It is active in esophageal squamous cell carcinoma [84], testicular germ cell tumors [85], ovarian cancer [86,87], and gastric cancer [88,89]. Vascular remodeling is a key feature of all these cancers. A large body of data suggests that tumor growth involves normal and abnormal vascular processes, nourishing tumor cells, and favoring their multiplication. Therefore, it is hardly surprising that the growth of human uveal melanomas is associated with abnormal vascularization processes. An ability to form vascular loops has recently been identified as an important prognostic factor in choroidal melanoma. Tumors may develop an intricate pattern of microcirculation independent of angiogenesis. In aggressive primary and metastatic melanomas, the tumor cells generate acellular microcirculatory channels composed of extracellular matrix and lined externally by tumor cells. The de novo generation of vascular channels by aggressive and metastatic tumor cells is not strictly vasculogenic because true vasculogenesis results in the de novo formation of endothelial cell-lined vessels. This “vasculogenic mimicry” allows aggressive tumor cells to generate non-endothelial cell-lined channels delimited by the extracellular matrix [90]. These cells produce vascular endothelium-cadherin (VE-cadherin), express the vascular endothelial growth factor (VEGF) receptor, and have high levels of metalloproteinase activity [91]. The acquisition of a more classical angiogenic phenotype is also required for the malignant progression of various solid tumors. Previous studies have demonstrated that several genes including those playing an important role in angiogenesis are differentially expressed in human melanoma cells [92]. Further characterization of the molecular mechanisms and transcription factors involved in the formation of this type of tumor may lead to the development of alternative methods of inhibiting or blocking tumor growth and/or interfering with metastasis.

ETS-1 is a critical regulator of Ang II-mediated vascular remodeling. Zhan et al. [38] identified several ETS-1 target gene products (including PAI-1 and MCP-1) involved in this pathway. PAI-1 is a major regulator of tumor invasion, metastasis, and cancer-related angiogenesis [51]. PAI-1 may interact with vitronectin, which normally promotes cell adhesion, spreading, and migration by interaction with integrins [93]. The coupling of PAI-1 to vitronectin prevents vitronectin-integrin interaction, which downregulates cell adhesion. Similarly, by competing with plasminogen activator, urokinase (uPA), the interaction of PAI-1 with vitronectin inhibits uPA-dependent cell adhesion. PAI-1 is also directly involved in tumorigenesis. Various cellular mechanisms contribute to PAI-1-regulated tumoral and choroidal neovascularization [94]. PAI-1 protects neovascularized tissues from excessive proteolysis [95] and controls in vivo tumor vascularization by interacting with proteases [96]. However, the upregulation of *PAI-1* mRNA levels may be part of a mechanism to protect the cell from destruction. Indeed, high levels of *PAI-1* expression are correlated with a poor prognosis in various types of cancer (gastric, breast, and lung) [97,98].

MCP-1 (or ccl2) is a chemokine that attracts and activates mononuclear cells. Many studies have shown that MCP-1 promotes tumor growth (for review see [50]). Cancer cells secrete chemokines to promote tumor growth and progression. For example, a high level of MCP-1 in breast cancer patients is associated with a significantly shorter relapse-free survival period than low levels of MCP-1 [99]. MCP-1 also seems to be involved in the recruitment of tumor-associated macrophages in several types of cancer (ovarian, gastric, breast, and esophageal) [100-103] and has pro-angiogenic activity [104]. The macrophages attracted by MCP-1 are potent sources of other angiogenic factors including VEGF. It therefore remains unclear whether the angiogenic effects of MCP-1 are direct or mediated by macrophage recruitment and activation [105]. MCP-1 seems to have a direct effect on tumors through its effects on angiogenesis [106]. MCP-1-deficient mice were shown to be protected in models of endogenous carcinoma development [50].

The p16INK4A cyclin-dependent kinase inhibitor has been implicated in replicative senescence, the state of permanent growth arrest induced by cumulative cell divisions or constitutive Ras-Raf-MEK signaling in somatic cells. Ohtani et al. [107] demonstrated a role for ETS-1 and ETS-2 in the regulation of p16INK4A production involving the binding to and activation of the *p16INK4A* gene promoter. We observed the upregulation of the *p16* gene expression in our model, consistent with the high levels of p16 detected in a rapidly growing malignant uveal melanoma in a previous case study [108]. However, other groups have found that *p16* gene expression levels in melanoma cells are lower than or the same as in normal cells. Similar to our findings, a previous study demonstrated an upregulation of *p16* in uveal melanoma [109]. They observed upregulation of cyclin D1, cyclin E, and p16INK4A, together with abnormal pRB and E2F binding. They concluded that the overproduction of cyclins D1 and E and Cyclin Dependant Kinases Inhibitor (CDKI) p16 together with the deregulation of the Rb/E2F pathway may be involved in the development of human uveal melanoma. Consistent with this, we observed the upregulation of *p16* in a mouse model of choroidal melanoma in which tumor formation is induced by the SV-40 T antigen. The gene encoding pRb is a target of several transforming viral oncoproteins including the T antigen of SV40 [110]. The upregulation of *p16* may also be due to the upregulation of the microphthalmia-associated transcription factor (MITF) transcription factor. Like ETS-1, MITF activates *p16* gene expression [111]. MITF has been described as a highly sensitive immunohistochemical marker for melanoma diagnosis. *MITF* gene amplification is involved in melanoma progression.

We then studied the expression level of the ETS-2 target gene encoding ICAM-1. *ICAM-1* mRNA levels were higher in this mouse model of choroidal melanoma than in controls. The gene encoding ICAM-1 is transactivated directly by ETS-2 [45,112]. ICAM-1 regulates cell–cell and cell–matrix adhesion, and its role in inflammation has been studied in detail [113]. Recent work based on the use of a mouse antibody against ICAM-1 has shown that blocking this adhesion molecule inhibits the growth of uveal melanoma in a severe combined immunideficient (SCID) mouse model [46]. A previous study revealed very high levels of *ICAM-1* gene expression in melanomas [114]. ICAM-1 acts in association with neutrophils by inducing polymorphonuclear (PMN) cell degranulation and releasing proteases, which break down the endothelial barrier and promote tumor cell migration during metastasis formation [115]. Our data are consistent with previous findings. Thus, upregulation of the production and activity of the ETS-2 transcription factor may promote cell proliferation and metastasis in this tumor model.

The changes observed in expression levels of ETS target genes seem modest compared to those of *ETS-1* and *ETS-2*. This may be due to the involvement of other transcription factors or transcription regulators possibly acting together at the promoters of these genes.

ETS-1 and/or ETS-2 could also be involved in epithelial-mesenchyme transitions (EMT). These transitions include a variety of intercellular and intracellular changes [116]. EMT plays an important role in the development of many tissues during embryogenesis, but similar cell changes occur during pathological processes such as cancer development. ETS-1 is produced during EMT [117]. Further experiments are required to confirm the involvement of ETS-1 and ETS-2 in this process.

In conclusion, this study shows that ETS-1 and ETS-2 may play a major role in choroidal melanoma. We have characterized the distribution of these two transcription factors in the normal and diseased eye. The upregulation of these proteins was correlated with upregulation of their target genes in a mouse model of ocular neoplasm. Thus, ETS-1 and ETS-2 may be involved in the development of this disease and are therefore potential targets for choroidal melanoma gene therapy. The next step could be to explore the consequences of an upregulation of these ETS transcription factors in normal human melanocytes to observe if these cells become neoplastics. Our findings are also consistent with those obtained in the ETS-1-dependent vascular remodeling model [38], which identifies ETS-1 as a direct target of Ang II in vascular remodeling mediated by MCP-1 and PAI-1. This ETS transcription factor, which increases the production of MCP-1 and PAI-1, both of which are involved in vascular remodeling, was significantly upregulated in our model. Our findings may have important implications for the development of new therapeutic agents for use in uveal melanoma. Indeed, vascular remodeling is known to be a key factor in the development of resistance to antiangiogenic treatment in tumors [118]. Future studies may confirm that vascular remodeling does indeed occur in this tumor. ETS-1 and angiotensin II receptors would thus be very important targets for cancer treatment. The study of human choroidal melanoma tumors should shed light on the role of the genes encoding ETS-1 and ETS-2 in the pathogenesis and progression of these tumors.

## References

[1] Moran E (1993). DNA tumor virus transforming proteins and the cell cycle.. Curr Opin Genet Dev.

[2] Ali SH, DeCaprio JA (2001). Cellular transformation by SV40 large T antigen: interaction with host proteins.. Semin Cancer Biol.

[3] Levine AJ (1990). The p53 protein and its interactions with the oncogene products of the small DNA tumor viruses.. Virology.

[4] DeCaprio JA, Ludlow JW, Figge J, Shew JY, Huang CM, Lee WH, Marsilio E, Paucha E, Livingston DM (1988). SV40 large tumor antigen forms a specific complex with the product of the retinoblastoma susceptibility gene.. Cell.

[5] Eckner R, Ludlow JW, Lill NL, Oldread E, Arany Z, Modjtahedi N, DeCaprio JA, Livingston DM, Morgan JA (1996). Association of p300 and CBP with simian virus 40 large T antigen.. Mol Cell Biol.

[6] Ahuja D, Saenz-Robles MT, Pipas JM (2005). SV40 large T antigen targets multiple cellular pathways to elicit cellular transformation.. Oncogene.

[7] Hahn WC, Counter CM, Lundberg AS, Beijersbergen RL, Brooks MW, Weinberg RA (1999). Creation of human tumour cells with defined genetic elements.. Nature.

[8] Bradl M, Klein-Szanto A, Porter S, Mintz B (1991). Malignant melanoma in transgenic mice.. Proc Natl Acad Sci USA.

[9] Syed NA, Windle JJ, Darjatmoko SR, Lokken JM, Steeves RA, Chappell R, Wallow IH, Koop BA, Mangold G, Howes KA, Albert DM (1998). Transgenic mice with pigmented intraocular tumors: tissue of origin and treatment.. Invest Ophthalmol Vis Sci.

[10] Penna D, Schmidt A, Beermann F (1998). Tumors of the retinal pigment epithelium metastasize to inguinal lymph nodes and spleen in tyrosinase-related protein 1/SV40 T antigen transgenic mice.. Oncogene.

[11] Larue L, Dougherty N, Bradl M, Mintz B (1993). Melanocyte culture lines from Tyr-SV40E transgenic mice: models for the molecular genetic evolution of malignant melanoma.. Oncogene.

[12] Larue L, Dougherty N, Mintz B (1992). Genetic predisposition of transgenic mouse melanocytes to melanoma results in malignant melanoma after exposure to a low ultraviolet B intensity nontumorigenic for normal melanocytes.. Proc Natl Acad Sci USA.

[13] Klein-Szanto A, Bradl M, Porter S, Mintz B (1991). Melanosis and associated tumors in transgenic mice.. Proc Natl Acad Sci USA.

[14] Murisier F, Guichard S, Beermann F (2006). A conserved transcriptional enhancer that specifies Tyrp1 expression to melanocytes.. Dev Biol.

[15] Sementchenko VI, Watson DK (2000). Ets target genes: past, present and future.. Oncogene.

[16] Sharrocks AD, Brown AL, Ling Y, Yates PR (1997). The ETS-domain transcription factor family.. Int J Biochem Cell Biol.

[17] Seth A, Watson DK (2005). ETS transcription factors and their emerging roles in human cancer.. Eur J Cancer.

[18] Albagli O, Soudant N, Ferreira E, Dhordain P, Dewitte F, Begue A, Flourens A, Stehelin D, Leprince D (1994). A model for gene evolution of the ets-1/ets-2 transcription factors based on structural and functional homologies.. Oncogene.

[19] Lautenberger JA, Burdett LA, Gunnell MA, Qi S, Watson DK, O'Brien SJ, Papas TS (1992). Genomic dispersal of the ets gene family during metazoan evolution.. Oncogene.

[20] Macleod K, Leprince D, Stehelin D (1992). The ets gene family.. Trends Biochem Sci.

[21] Dittmer J (2003). The biology of the Ets1 proto-oncogene.. Mol Cancer.

[22] Keehn CA, Smoller BR, Morgan MB (2003). Expression of the ets-1 proto-oncogene in melanocytic lesions.. Mod Pathol.

[23] Takai N, Miyazaki T, Nishida M, Nasu K, Miyakawa I (2002). c-Ets1 is a promising marker in epithelial ovarian cancer.. Int J Mol Med.

[24] Fujimoto J, Aoki I, Toyoki H, Khatun S, Tamaya T (2002). Clinical implications of expression of ETS-1 related to angiogenesis in uterine cervical cancers.. Ann Oncol.

[25] Span PN, Manders P, Heuvel JJ, Thomas CM, Bosch RR, Beex LV, Sweep CG (2002). Expression of the transcription factor Ets-1 is an independent prognostic marker for relapse-free survival in breast cancer.. Oncogene.

[26] Pande P, Mathur M, Shukla NK, Ralhan R (1999). Ets-1: a plausible marker of invasive potential and lymph node metastasis in human oral squamous cell carcinomas.. J Pathol.

[27] Sasaki H, Yukiue H, Moiriyama S, Kobayashi Y, Nakashima Y, Kaji M, Kiriyama M, Fukai I, Yamakawa Y, Fujii Y (2001). Clinical significance of matrix metalloproteinase-7 and Ets-1 gene expression in patients with lung cancer.. J Surg Res.

[28] Sacchi N, de Klein A, Showalter SD, Bigi G, Papas TS (1988). High expression of ets-1 gene in human thymocytes and immature T leukemic cells.. Leukemia.

[29] Nishikawa A, Iwasaki M, Akutagawa N, Manase K, Yamashita S, Endo T, Kudo R (2000). Expression of various matrix proteases and Ets family transcriptional factors in ovarian cancer cell lines: correlation to invasive potential.. Gynecol Oncol.

[30] Naito S, Shimizu K, Nakashima M, Nakayama T, Ito T, Ito M, Yamashita S, Sekine I (2000). Overexpression of Ets-1 transcription factor in angiosarcoma of the skin.. Pathol Res Pract.

[31] Duda DG, Sunamura M, Lefter LP, Furukawa T, Yokoyama T, Yatsuoka T, Abe T, Inoue H, Motoi F, Egawa S, Matsuno S, Horii A (2003). Restoration of SMAD4 by gene therapy reverses the invasive phenotype in pancreatic adenocarcinoma cells.. Oncogene.

[32] Pham VN, Lawson ND, Mugford JW, Dye L, Castranova D, Lo B, Weinstein BM (2007). Combinatorial function of ETS transcription factors in the developing vasculature.. Dev Biol.

[33] Wernert N, Stanjek A, Kiriakidis S, Hugel A, Jha HC, Mazitschek R, Giannis A (1999). Inhibition of Angiogenesis In Vivo by ets-1 Antisense Oligonucleotides-Inhibition of Ets-1 Transcription Factor Expression by the Antibiotic Fumagillin.. Angew Chem Int Ed Engl.

[34] Nakano T, Abe M, Tanaka K, Shineha R, Satomi S, Sato Y (2000). Angiogenesis inhibition by transdominant mutant Ets-1.. J Cell Physiol.

[35] Barton K, Muthusamy N, Fischer C, Ting CN, Walunas TL, Lanier LL, Leiden JM (1998). The Ets-1 transcription factor is required for the development of natural killer cells in mice.. Immunity.

[36] Bories JC, Willerford DM, Grevin D, Davidson L, Camus A, Martin P, Stehelin D, Alt FW (1995). Increased T-cell apoptosis and terminal B-cell differentiation induced by inactivation of the Ets-1 proto-oncogene.. Nature.

[37] Muthusamy N, Barton K, Leiden JM (1995). Defective activation and survival of T cells lacking the Ets-1 transcription factor.. Nature.

[38] Zhan Y, Brown C, Maynard E, Anshelevich A, Ni W, Ho IC, Oettgen P (2005). Ets-1 is a critical regulator of Ang II-mediated vascular inflammation and remodeling.. J Clin Invest.

[39] Wolvetang EW, Bradfield OM, Tymms M, Zavarsek S, Hatzistavrou T, Kola I, Hertzog PJ (2003). The chromosome 21 transcription factor ETS2 transactivates the beta-APP promoter: implications for Down syndrome.. Biochim Biophys Acta.

[40] Sanij E, Hatzistavrou T, Hertzog P, Kola I, Wolvetang EJ (2001). Ets-2 is induced by oxidative stress and sensitizes cells to H(2)O(2)-induced apoptosis: implications for Down's syndrome.. Biochem Biophys Res Commun.

[41] Wolvetang EJ, Bradfield OM, Hatzistavrou T, Crack PJ, Busciglio J, Kola I, Hertzog PJ (2003). Overexpression of the chromosome 21 transcription factor Ets2 induces neuronal apoptosis.. Neurobiol Dis.

[42] Carbone GM, Napoli S, Valentini A, Cavalli F, Watson DK, Catapano CV (2004). Triplex DNA-mediated downregulation of Ets2 expression results in growth inhibition and apoptosis in human prostate cancer cells.. Nucleic Acids Res.

[43] Myers E, Hill AD, Kelly G, McDermott EW, O'Higgins NJ, Buggy Y, Young LS (2005). Associations and interactions between Ets-1 and Ets-2 and coregulatory proteins, SRC-1, AIB1, and NCoR in breast cancer.. Clin Cancer Res.

[44] Svensson S, Jirstrom K, Ryden L, Roos G, Emdin S, Ostrowski MC, Landberg G (2005). ERK phosphorylation is linked to VEGFR2 expression and Ets-2 phosphorylation in breast cancer and is associated with tamoxifen treatment resistance and small tumours with good prognosis.. Oncogene.

[45] de Launoit Y, Audette M, Pelczar H, Plaza S, Baert JL (1998). The transcription of the intercellular adhesion molecule-1 is regulated by Ets transcription factors.. Oncogene.

[46] Wang S, Coleman EJ, Pop LM, Brooks KJ, Vitetta ES, Niederkorn JY (2006). Effect of an anti-CD54 (ICAM-1) monoclonal antibody (UV3) on the growth of human uveal melanoma cells transplanted heterotopically and orthotopically in SCID mice.. Int J Cancer.

[47] Frau E, Magnon C, Opolon P, Connault E, Opolon D, Beermann F, Abitbol M, Perricaudet M, Bouquet C (2007). A gene transfer comparative study of HSA-conjugated antiangiogenic factors in a transgenic mouse model of metastatic ocular cancer.. Cancer Gene Ther.

[48] Young ID, Stewart RJ, Ailles L, Mackie A, Gore J (1993). Synthesis of digoxigenin-labeled cRNA probes for nonisotopic in situ hybridization using reverse transcription polymerase chain reaction.. Biotech Histochem.

[49] Suzuki T, Akimoto M, Mandai M, Takahashi M, Yoshimura N (2005). A new PCR-based approach for the preparation of RNA probe.. J Biochem Biophys Methods.

[50] Conti I, Rollins BJ (2004). CCL2 (monocyte chemoattractant protein-1) and cancer.. Semin Cancer Biol.

[51] Chorostowska-Wynimko J, Skrzypczak-Jankun E, Jankun J (2004). Plasminogen activator inhibitor type-1: its structure, biological activity and role in tumorigenesis. Int J Mol Med.

[52] McCabe KL, McGuire C, Reh TA (2006). Pea3 expression is regulated by FGF signaling in developing retina.. Dev Dyn.

[53] Jobling AI, Fang Z, Koleski D, Tymms MJ (2002). Expression of the ETS transcription factor ELF3 in the retinal pigment epithelium.. Invest Ophthalmol Vis Sci.

[54] Yoshida N, Yoshida S, Araie M, Handa H, Nabeshima Y (2000). Ets family transcription factor ESE-1 is expressed in corneal epithelial cells and is involved in their differentiation.. Mech Dev.

[55] Treier M, Bohmann D, Mlodzik M (1995). JUN cooperates with the ETS domain protein pointed to induce photoreceptor R7 fate in the Drosophila eye.. Cell.

[56] O'Neill EM, Rebay I, Tjian R, Rubin GM (1994). The activities of two Ets-related transcription factors required for Drosophila eye development are modulated by the Ras/MAPK pathway.. Cell.

[57] Brunner D, Ducker K, Oellers N, Hafen E, Scholz H, Klambt C (1994). The ETS domain protein pointed-P2 is a target of MAP kinase in the sevenless signal transduction pathway.. Nature.

[58] Fujiwara S, Fisher RJ, Seth A, Bhat NK, Showalter SD, Zweig M, Papas TS (1988). Characterization and localization of the products of the human homologs of the v-ets oncogene.. Oncogene.

[59] Maroulakou IG, Papas TS, Green JE (1994). Differential expression of ets-1 and ets-2 proto-oncogenes during murine embryogenesis.. Oncogene.

[60] Gilliland DG (2001). The diverse role of the ETS family of transcription factors in cancer.. Clin Cancer Res.

[61] Oikawa T (2004). ETS transcription factors: possible targets for cancer therapy.. Cancer Sci.

[62] Yang ZF, Mott S, Rosmarin AG (2007). The Ets transcription factor GABP is required for cell-cycle progression.. Nat Cell Biol.

[63] Cavender JF, Conn A, Epler M, Lacko H, Tevethia MJ (1995). Simian virus 40 large T antigen contains two independent activities that cooperate with a ras oncogene to transform rat embryo fibroblasts.. J Virol.

[64] Sachsenmeier KF, Pipas JM (2001). Inhibition of Rb and p53 is insufficient for SV40 T-antigen transformation.. Virology.

[65] Valls E, Blanco-Garcia N, Aquizu N, Piedra D, Estaras C, de la Cruz X, Martinez-Balbas MA (2007). Involvement of chromatin and histone deacetylation in SV40 T antigen transcription regulation.. Nucleic Acids Res.

[66] Rhee I, Bachman KE, Park BH, Jair KW, Yen RW, Schuebel KE, Cui H, Feinberg AP, Lengauer C, Kinzler KW, Baylin SB, Vogelstein B (2002). DNMT1 and DNMT3b cooperate to silence genes in human cancer cells.. Nature.

[67] Herman JG, Baylin SB (2003). Gene silencing in cancer in association with promoter hypermethylation.. N Engl J Med.

[68] Robert MF, Morin S, Beaulieu N, Gauthier F, Chute IC, Barsalou A, MacLeod AR (2003). DNMT1 is required to maintain CpG methylation and aberrant gene silencing in human cancer cells.. Nat Genet.

[69] Luczak MW, Jagodzinski PP (2006). The role of DNA methylation in cancer development.. Folia Histochem Cytobiol.

[70] Hadnagy A, Beaulieu R, Balicki D (2008). Histone tail modifications and noncanonical functions of histones: perspectives in cancer epigenetics.. Mol Cancer Ther.

[71] Onken MD, Ehlers JP, Worley LA, Makita J, Yokota Y, Harbour JW (2006). Functional gene expression analysis uncovers phenotypic switch in aggressive uveal melanomas.. Cancer Res.

[72] Gambrelle J, Labialle S, Dayan G, Gayet L, Barakat S, Michaud M, Grange JD, Baggetto LG (2005). Multidrug resistance in uveal melanoma.. J Fr Ophtalmol.

[73] Labialle S, Dayan G, Gambrelle J, Gayet L, Barakat S, Devouassoux-Shisheboran M, Bernaud J, Rigal D, Grange JD, Baggetto LG (2005). Characterization of the typical multidrug resistance profile in human uveal melanoma cell lines and in mouse liver metastasis derivatives.. Melanoma Res.

[74] Kida Y, Han YP (2008). MicroRNA expression in colon adenocarcinoma.. JAMA.

[75] Schetter AJ, Leung SY, Sohn JJ, Zanetti KA, Bowman ED, Yanaihara N, Yuen ST, Chan TL, Kwong DL, Au GK, Liu CG, Calin GA, Croce CM, Harris CC (2008). MicroRNA expression profiles associated with prognosis and therapeutic outcome in colon adenocarcinoma.. JAMA.

[76] Wu TT, Hsieh YH, Hsieh YS, Liu JY (2008). Reduction of PKC alpha decreases cell proliferation, migration, and invasion of human malignant hepatocellular carcinoma.. J Cell Biochem.

[77] La Porta CA, Di Dio A, Comolli R (1999). Inhibition of PKCalpha decreases the gelatinase activity and the angiogenic and metastatic ability of the highly metastatic B16 murine melanoma cells.. Angiogenesis.

[78] Zhao X, Murata T, Ohno S, Day N, Song J, Nomura N, Nakahara T, Yokoyama KK (2001). Protein kinase Calpha plays a critical role in mannosylerythritol lipid-induced differentiation of melanoma B16 cells.. J Biol Chem.

[79] Sullivan RM, Stone M, Marshall JF, Uberall F, Rotenberg SA (2000). Photo-induced inactivation of protein kinase calpha by dequalinium inhibits motility of murine melanoma cells.. Mol Pharmacol.

[80] Vetter M, Blumenthal SG, Lindemann RK, Manns J, Wesselborg S, Thomssen C, Dittmer J (2005). Ets1 is an effector of protein kinase Calpha in cancer cells.. Oncogene.

[81] Foulds CE, Nelson ML, Blaszczak AG, Graves BJ (2004). Ras/mitogen-activated protein kinase signaling activates Ets-1 and Ets-2 by CBP/p300 recruitment.. Mol Cell Biol.

[82] Pufall MA, Lee GM, Nelson ML, Kang HS, Velyvis A, Kay LE, McIntosh LP, Graves BJ (2005). Variable control of Ets-1 DNA binding by multiple phosphates in an unstructured region.. Science.

[83] Cowley DO, Graves BJ (2000). Phosphorylation represses Ets-1 DNA binding by reinforcing autoinhibition.. Genes Dev.

[84] Mukherjee T, Kumar A, Mathur M, Chattopadhyay TK, Ralhan R (2003). Ets-1 and VEGF expression correlates with tumor angiogenesis, lymph node metastasis, and patient survival in esophageal squamous cell carcinoma.. J Cancer Res Clin Oncol.

[85] Adam M, Schmidt D, Wardelmann E, Wernert N, Albers P (2003). Angiogenetic protooncogene ets-1 induced neovascularization is involved in the metastatic process of testicular germ cell tumors.. Eur Urol.

[86] Khatun S, Fujimoto J, Toyoki H, Tamaya T (2003). Clinical implications of expression of ETS-1 in relation to angiogenesis in ovarian cancers.. Cancer Sci.

[87] Fujimoto J, Aoki I, Toyoki H, Khatun S, Sato E, Sakaguchi H, Tamaya T (2004). Clinical implications of expression of ETS-1 related to angiogenesis in metastatic lesions of ovarian cancers.. Oncology.

[88] Tsutsumi S, Kuwano H, Nagashima N, Shimura T, Mochiki E, Asao T (2005). Ets-1 expression in gastric cancer.. Hepatogastroenterology.

[89] Tsutsumi S, Kuwano H, Asao T, Nagashima K, Shimura T, Mochiki E (2000). Expression of Ets-1 angiogenesis-related protein in gastric cancer.. Cancer Lett.

[90] Folberg R, Hendrix MJ, Maniotis AJ (2000). Vasculogenic mimicry and tumor angiogenesis.. Am J Pathol.

[91] Hendrix MJ, Seftor EA, Meltzer PS, Gardner LM, Hess AR, Kirschmann DA, Schatteman GC, Seftor RE (2001). Expression and functional significance of VE-cadherin in aggressive human melanoma cells: role in vasculogenic mimicry.. Proc Natl Acad Sci USA.

[92] Albert D, Polans A. Ocular Oncology. New York: Marcel Dekker; 2003.

[93] Schvartz I, Seger D, Shaltiel S (1999). Vitronectin.. Int J Biochem Cell Biol.

[94] Jost M, Maillard C, Lecomte J, Lambert V, Tjwa M, Blaise P, Alvarez Gonzalez ML, Bajou K, Blacher S, Motte P, Humblet C, Defresne MP, Thiry M, Frankenne F, Gothot A, Carmeliet P, Rakic JM, Foidart JM, Noel A (2007). Tumoral and choroidal vascularization: differential cellular mechanisms involving plasminogen activator inhibitor type I.. Am J Pathol.

[95] Bacharach E, Itin A, Keshet E (1992). In vivo patterns of expression of urokinase and its inhibitor PAI-1 suggest a concerted role in regulating physiological angiogenesis.. Proc Natl Acad Sci USA.

[96] Bajou K, Masson V, Gerard RD, Schmitt PM, Albert V, Praus M, Lund LR, Frandsen TL, Brunner N, Dano K, Fusenig NE, Weidle U, Carmeliet G, Loskutoff D, Collen D, Carmeliet P, Foidart JM, Noel A (2001). The plasminogen activator inhibitor PAI-1 controls in vivo tumor vascularization by interaction with proteases, not vitronectin. Implications for antiangiogenic strategies.. J Cell Biol.

[97] Kaneko T, Konno H, Baba M, Tanaka T, Nakamura S (2003). Urokinase-type plasminogen activator expression correlates with tumor angiogenesis and poor outcome in gastric cancer.. Cancer Sci.

[98] Gershtein ES, Kushlinskii NE (2001). Urokinase and tissue plasminogen activators and their inhibitor PAI-1 in human tumors.. Bull Exp Biol Med.

[99] Ueno T, Toi M, Saji H, Muta M, Bando H, Kuroi K, Koike M, Inadera H, Matsushima K (2000). Significance of macrophage chemoattractant protein-1 in macrophage recruitment, angiogenesis, and survival in human breast cancer.. Clin Cancer Res.

[100] Negus RP, Stamp GW, Hadley J, Balkwill FR (1997). Quantitative assessment of the leukocyte infiltrate in ovarian cancer and its relationship to the expression of C–C chemokines.. Am J Pathol.

[101] Leek RD, Lewis CE, Whitehouse R, Greenall M, Clarke J, Harris AL (1996). Association of macrophage infiltration with angiogenesis and prognosis in invasive breast carcinoma.. Cancer Res.

[102] Ohta M, Kitadai Y, Tanaka S, Yoshihara M, Yasui W, Mukaida N, Haruma K, Chayama K (2003). Monocyte chemoattractant protein-1 expression correlates with macrophage infiltration and tumor vascularity in human gastric carcinomas.. Int J Oncol.

[103] Ohta M, Kitadai Y, Tanaka S, Yoshihara M, Yasui W, Mukaida N, Haruma K, Chayama K (2002). Monocyte chemoattractant protein-1 expression correlates with macrophage infiltration and tumor vascularity in human esophageal squamous cell carcinomas.. Int J Cancer.

[104] Salcedo R, Ponce ML, Young HA, Wasserman K, Ward JM, Kleinman HK, Oppenheim JJ, Murphy WJ (2000). Human endothelial cells express CCR2 and respond to MCP-1: direct role of MCP-1 in angiogenesis and tumor progression.. Blood.

[105] Goede V, Brogelli L, Ziche M, Augustin HG (1999). Induction of inflammatory angiogenesis by monocyte chemoattractant protein-1.. Int J Cancer.

[106] Stamatovic SM, Keep RF, Mostarica-Stojkovic M, Andjelkovic AV (2006). CCL2 regulates angiogenesis via activation of Ets-1 transcription factor.. J Immunol.

[107] Ohtani N, Zebedee Z, Huot TJ, Stinson JA, Sugimoto M, Ohashi Y, Sharrocks AD, Peters G, Hara E (2001). Opposing effects of Ets and Id proteins on p16INK4a expression during cellular senescence.. Nature.

[108] Honda S, Hirai T, Handa JT, Okuno T, Saito I (2004). Expression of cell cycle related proteins in a rapidly growing uveal malignant melanoma.. Retina.

[109] Pardo M, Pineiro A, de la Fuente M, Garcia A, Prabhakar S, Zitzmann N, Dwek RA, Sanchez-Salorio M, Dominguez F, Capeans C (2004). Abnormal cell cycle regulation in primary human uveal melanoma cultures.. J Cell Biochem.

[110] Dyson N, Buchkovich K, Whyte P, Harlow E (1989). Cellular proteins that are targetted by DNA tumor viruses for transformation.. Princess Takamatsu Symp.

[111] Loercher AE, Tank EM, Delston RB, Harbour JW (2005). MITF links differentiation with cell cycle arrest in melanocytes by transcriptional activation of INK4A.. J Cell Biol.

[112] Roy J, Audette M, Tremblay MJ (2001). Intercellular adhesion molecule-1 (ICAM-1) gene expression in human T cells is regulated by phosphotyrosyl phosphatase activity. Involvement of NF-kappaB, Ets, and palindromic interferon-gamma-responsive element-binding sites.. J Biol Chem.

[113] Dustin ML, Rothlein R, Bhan AK, Dinarello CA, Springer TA (1986). Induction by IL 1 and interferon-gamma: tissue distribution, biochemistry, and function of a natural adherence molecule (ICAM-1).. J Immunol.

[114] Johnson JP, Stade BG, Holzmann B, Schwable W, Riethmuller G (1989). De novo expression of intercellular-adhesion molecule 1 in melanoma correlates with increased risk of metastasis.. Proc Natl Acad Sci USA.

[115] Barnett CC, Moore EE, Mierau GW, Partrick DA, Biffl WL, Elzi DJ, Silliman CC (1998). ICAM-1–CD18 interaction mediates neutrophil cytotoxicity through protease release.. Am J Physiol.

[116] Thiery JP, Sleeman JP (2006). Complex networks orchestrate epithelial-mesenchymal transitions.. Nat Rev Mol Cell Biol.

[117] Fafeur V, Tulasne D, Queva C, Vercamer C, Dimster V, Mattot V, Stehelin D, Desbiens X, Vandenbunder B (1997). The ETS1 transcription factor is expressed during epithelial-mesenchymal transitions in the chick embryo and is activated in scatter factor-stimulated MDCK epithelial cells.. Cell Growth Differ.

[118] Glade Bender J, Cooney EM, Kandel JJ, Yamashiro DJ (2004). Vascular remodeling and clinical resistance to antiangiogenic cancer therapy.. Drug Resist Updat.

